# The role of Trithorax family regulating osteogenic and Chondrogenic differentiation in mesenchymal stem cells

**DOI:** 10.1111/cpr.13233

**Published:** 2022-04-28

**Authors:** Qingge Ma, Chenghao Song, Bei Yin, Yu Shi, Ling Ye

**Affiliations:** ^1^ State Key Laboratory of Oral Diseases & National Clinical Research Center for Oral Diseases, West China Hospital of Stomatology Sichuan University Chengdu China; ^2^ Department of Endodontics, West China Hospital of Stomatology Sichuan University Chengdu China

## Abstract

Mesenchymal stem/stromal cells (MSCs) hold great promise and clinical efficacy in bone/cartilage regeneration. With a deeper understanding of stem cell biology over the past decade, epigenetics stands out as one of the most promising ways to control MSCs differentiation. Trithorax group (TrxG) proteins, including the COMPASS family, ASH1L, CBP/p300 as histone modifying factors, and the SWI/SNF complexes as chromatin remodelers, play an important role in gene expression regulation during the process of stem cell differentiation. This review summarises the components and functions of TrxG complexes. We provide an overview of the regulation mechanisms of TrxG in MSCs osteogenic and chondrogenic differentiation, and discuss the prospects of epigenetic regulation mediated by TrxG in bone and cartilage regeneration.

## Graphical abstract

TrxG family is one of the most important mechanisms for gene expression activation in epigenetics. The COMPASS family, ASH1L, and CBP/p300 are associated with histone methylation and acetylation activities. The SWI/SNF complexes have ATP‐dependent chromatin remodelling activities. By targeting differentiation‐related key genes such as *RUNX2*, *OSX*, *DLX3*, and *SOX9*, these TrxG proteins play a prominent role in the epigenetic regulation of osteogenic and chondrogenic differentiation.

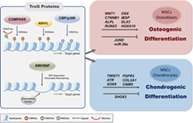

## 1 INTRODUCTION

Bone is a metabolically active and dynamic tissue with the capability of rapid remodelling to heal defects smaller than critical size perfectly. Bone regeneration is required under various physiological and pathological situations that cause bone loss including trauma, infection, skeletal abnormality or tumour.^1^ But bone cannot heal itself perfectly in the case of critical bone defects. Cartilage is a resilient connective tissue that functions as supportive and conjunctive components of the body. Opposite to the abundant vascular in bone, cartilage is avascular and aneural, thus it has limited regeneration capabilities. Even small cartilage defects may require surgical intervention.^2^ Treatments of bone and cartilage diseases such as osteoporosis (OP) and osteoarthritis (OA) require precise regulation from system and organic level to cellular and molecular level. Significant efforts have been made in recent years in the development of stem cell transplantation for bone/cartilage repair.^3^, ^4^ Among the various stem cell sources, as one of the most important participants of bone/cartilage healing process, mesenchymal stem/stromal cells (MSCs) have gained increasing focus, holding great promise, and clinical efficacy in bone/cartilage regeneration. MSCs have self‐renewal abilities and multi‐differentiation potential towards osteoblasts, chondrocytes, adipocytes, etc. The differentiation direction of MSCs is affected by various transcription factors and growth factors including runt‐related transcription factor 2 (RUNX2), sp7 transcription factor (SP7; also named as OSX), SRY‐box transcription factor 9 (SOX9), and bone morphogenetic protein 2 (BMP2).^5^, ^6^, ^7^ With a deeper understanding of stem cell biology, epigenetics stands out as one of the most promising ways to control MSCs differentiation.

There is mounting evidence suggesting that epigenetic regulation of gene expression plays an essential role in stem cell fate determination during development.^8^, ^9^ Epigenetic modifications occur on the chromatin level without changing the DNA sequence. DNA methylation and histone modifications are key epigenetic patterns that play extensive roles in gene regulation.^10^ Concerning histone modification, the most studied epigenetic factors over decades are Polycomb group (PcG) and Trithorax group (TrxG) of proteins. Initially discovered in Drosophila as heterogeneous groups of factors, PcG and TrxG proteins have antagonistic roles in transcriptional regulations of homeotic (*HOX*) genes and other target genes.^11^ The Trithorax proteins activate gene expression and counteract PcG‐mediated transcriptional repression by modifying chromatin through their histone methyltransferase or acetyltransferase activities. They are profoundly involved in stem cell proliferation and differentiation.^12^ In this review, we briefly introduce the composition, biological function, and regulation mechanisms of TrxG proteins, and discuss the regulatory role of TrxG proteins in MSCs osteogenic and chondrogenic differentiation.

## 2 THE COMPOSITION OF TRXG COMPLEXES

TrxG proteins mediate epigenetic activation in the form of both complexes (COMPASS family and SWI/SNF family) and individual proteins (ASH1L and CBP/p300).^13^, ^14^ The Complex of Proteins Associated with Set1 (COMPASS) family is well‐known for its histone H3 lysine 4 (H3K4) methyltransferase activity with a highly conserved protein domain, Suppressor of variegation 3–9, Enhancer of zeste, Trithorax (SET).^15^ Human COMPASS family consists of six complexes that can be divided into three subtypes, SET1/COMPASS, MLL1/2 COMPASS‐like, and MLL3/4 COMPASS‐like, each containing a SET‐domain methyltransferase as central catalytic subunit.^12^ All the six complexes have a common core structure consisting of 4 proteins: WD repeat‐containing protein 5 (WDR5), retinoblastoma‐binding protein 5 (RBBP5), absent, small, or homeotic‐like2 (ASH2L), and dumpy‐30 (DPY30), short as “WRAD” or “WARD”.^16^ SET1/COMPASS complexes are responsible for global H3K4 trimethylation (H3K4me3) in cells, and addedly contain host cell factor C1 (HCFC1), WD repeat‐containing protein 82 (WDR82), CXXC‐type zinc finger protein 1 (CFP1), and either SET domain containing 1A (SETD1A) or SET domain containing 1B (SETD1B).^17^, ^18^ The mixed lineage leukaemia (MLL) proteins are SET‐domain containing lysine methyltransferases (KMTs) functioning as core catalytic member in four COMPASS‐like complexes, respectively. MLL1/2 COMPASS‐like additionally contains menin 1 (MEN1) and HCFC1, while MLL3/4 COMPASS‐like additionally contains lysine demethylase 6A (KDM6A), nuclear receptor coactivator 6 (NCOA6), PAXIP1‐associated glutamate‐rich protein 1 (PAGR1), and PAX‐interacting protein 1 (PAXIP1).^10^

Another group of TrxG complexes has ATP‐dependent chromatin‐remodelling activities. The SWI/SNF (switching/sucrose non‐fermentable) complex genes were identified to have counteractive roles against Polycomb‐mediated repression of HOX genes in Drosophila.^14^ The mammalian homologues of SWI/SNF, consisting of approximately 10 subunits, contain either SMARCA4 (BRG1) or SMARCA2 (BRM) as an ATPase subunit, and exist in three structurally different forms: BRG1/BRM‐associated factors (BAFs), polybromo‐associated BAF complexes (PBAFs), and non‐canonical BAFs (ncBAFs).^19^

Other members of TrxG family have not been demonstrated to exist in stable complexes, though physical and functional interactions occur among proteins. The SET‐domain containing KMT, absent small or homeotic disc1 (ASH1), is reported to be associated with the histone acetyltransferase (HAT) CREB binding protein (CBP) in Drosophila.^20^ Human ASH1 homologue absent small or homeotic disc1 like (ASH1L) is reported to function within a protein complex that consists of MRG domain‐containing proteins MORF4L1 and MORF4L2, and WD40 domain‐containing proteins RBBP7 and RBBP4.^21^ The mammalian CREB binding protein (CREBBP) and its paralogue E1A binding protein p300 (EP300) are important transcriptional co‐activator involved in development.^22^

## 3 THE ROLE OF TRXG COMPONENTS IN OSTEOGENIC/CHONDROGENIC DIFFERENTIATIONS

### 3.1 COMPASS COMPLEXES

As a core subunit of COMPASS KMTs, WRAD is required for complex assembling and functioning. Structurally, DPY30 directly binds to ASH2L via a DPY‐30 binding‐motif (DBM) on the C‐terminus of ASH2L, while RBBP5 interacts with the SPRY domain of ASH2L and bridges it to WDR5.^23^, ^24^ The WRAD subcomplex possesses an H3K4 monomethylation activity independently and prefers histone H3/H4 tetramer instead of nucleosomal H3 as substrates. WDR5, RBBP5, and ASH2L form a minimal complex required for H3 methylation while DPY30 functions to increase complex stability, substrate specificity, and enzymatic activity.^25^, ^26^ In vitro experiments revealed that in the absence of WRAD, the SET domain of SETD1A protein is inactive and the MLL proteins merely have weak mono‐ or dimethyltransferases activities, suggesting that the interaction with WRAD subcomplex is crucial for COMPASS complexes canonical enzymatic functions.^27^

The regulatory role of WRAD subunits, especially WDR5, on stem cells differentiation has been well investigated. WDR5 belongs to a large protein family containing the conservative WD‐repeats domains and is involved in multiprotein complex assembling, localization and regulation.^16^, ^28^ The WD‐repeat β‐propeller structures of WDR5 interact with RBBP5 protein and the WDR5‐interacting (Win) motif of the six SET‐domain‐containing proteins, mediating COMPASS complexes assembling.^29^, ^30^ Moreover, WDR5 can directly bind the N‐terminal tail of histone H3 mediating enzyme–substrate interaction.^31^ Previous studies have demonstrated that WDR5 could be induced by BMP2 treatment in preosteoblasts and possessed an essential role in osteoblast differentiation via activation of the canonical Wnt signalling. Overexpression of WDR5 in MC3T3‐E1 osteoblastic cells promotes osteoblastic differentiation, whereas suppression of WDR5 expression dramatically impairs osteoblast differentiation. WDR5 was specifically recruited to the promoter of Wnt1 and Wnt downstream transcription factors myelocytomatosis oncogene (*Myc*) and *Runx2*.^32^, ^33^, ^34^ In calvarial cells isolated from *Osx* ^−/−^ mice, the occupancy of WDR5 and H3K4me3 level at the promoter of OSX target gene bone sialoprotein 2 (*Ibsp*) was significantly reduced, correlating with *Ibsp* expression decrease and repression of osteoblastic differentiation.^35^ A recent study revealed that a long non‐coding RNA (LncRNA) *HOTTIP* interacted with WDR5, and knockdown of *HOTTIP* impacted WDR5 nuclear translocation. WDR5 directly bound the promoter regions of catenin beta 1 (*CTNNB1*) mediating transcriptional activation. The *HOTTIP*‐WDR5 interaction activated Wnt/β‐catenin signalling during osteogenic differentiation of human bone marrow mesenchymal stem cells (hBMSCs).^36^

Furthermore, it has been reported that overexpression of WDR5 in mice perichondrium, under the control of collagen type I alpha 1 chain (*Col1a1*) promoter, caused a phenotype of larger skeleton, acceleration of endochondral and intramembranous bone formation, and increased hypertrophic chondrocyte layer size. In vitro experiments indicated that WDR5 enhanced expression of a perichondrium‐specific gene, twist basic helix–loop–helix transcription factor 1 (*Twist1*), leading to fibroblast growth factor 18 (*Fgf18*) downregulation, and resulting in promotion of chondrocyte proliferation and differentiation.^37^, ^38^, ^39^ Taken together, WDR5 exhibits a positive role in transcriptional regulation of genes involved in stem cells osteogenic and chondrogenic differentiation.

The two mammalian homologues of Drosophila Trithorax gene, *MLL1* (also known as *KMT2A*) and *MLL2* (*KMT2B*), are mutually exclusive in human MLL1/2 COMPASS‐like complex which additionally contains WRAD core structure, MEN1 and HCFC1.^40^, ^41^ MLL1 complex catalyses trimethylation of H3K4 at the promoters of less than 5% of genes including the *HOX* genes, while MLL2 is the major methyltransferase responsible for H3K4me3 on bivalent genes in mouse embryonic stem cells.^42^, ^43^ Despite the vast understanding of MLL1 and MLL2 functions in haematopoiesis and leukemogenesis, MLL1/2 COMPASS‐like also participates in skeleton development and regulation of osteogenesis and chondrogenesis. A previous study showed that transgenic mice expressing SET‐domain truncated MLL1 exhibited skeletal defects such as vertebral column and sternal malformations during development. ChIP analysis of the mutant embryos trunk sections revealed a significant decrease in H3K4me1 at *HOXD4* and *HOXC8* promoters, associated with reduced *HOX* gene expression.^44^ A member of *HOX* genes, distal‐less homeobox 3 (*DLX3*), promotes osteogenic differentiation of stem cells by targeting *RUNX2*. In vitro experiments in human dental follicle stem cells (DFCs) showed that activation of *DLX3* expression was regulated by MLL1/2 complexes and SUMO specific peptidase 3 (SENP3). Decreased deposition of MLL1/2 complexes components and reduction of H3K4me3 marks were found on the *DLX3* gene when *SENP3* was knockdown, leading to inhibited osteogenic differentiation of DFCs.^45^

Menin 1(MEN1) is considered as a tumour suppressor associated with multiple endocrine neoplasia type 1 sydrome.^46^ Over the decades, several researches have assessed the critical role of menin in bone development and bone metabolism. Homozygous *MEN1* gene knockout mice were found to be embryonic lethal and 20% of embryos showed cranial and facial developmental defects.^47^ *Men1* inactivation by antisense oligonucleotides (AS‐oligo) compromised the activity and expression of alkaline phosphatase (*Alpl*), *Col1a1*, *Runx2* and osteocalcin (*Bglap*) induced by BMP2 in C3H10T1/2 murine mesenchymal progenitor cells and ST2 stromal cells. Mechanistically, it was found that menin physically and functionally interacted with the BMP‐signalling downstream factors SMAD1, SMAD5, and RUNX2 in uncommitted MSCs and activated transcription of differentiation‐related genes.^48^, ^49^ Menin was reported to interact with Wnt‐signalling related factors, lymphoid enhancer binding factor 1 (LEF1) and β‐catenin, facilitated osteogenic differentiation via the canonical Wnt‐signalling pathway in murine myoblast cell line C2C12 cells.^50^ However, in well‐differentiated MC3T3‐E1 cells, menin interacted with transforming growth factor beta 1 (TGFB1) and SMAD3, resulting in suppression of BMP2‐induced transcriptional activities of SMAD1/5 and RUNX2. Inactivation of menin in MC3T3‐E1 cells increased ALP activity, mineralization, and the expression of *Col1a1* and *Bglap*.^48^, ^49^ In addition, menin was co‐immunoprecipitated with JUND, the activator protein‐1 transcription factor subunit, when co‐transfected into MC3T3‐E1 cells, and suppressed JUND induced osteoblasts maturation.^51^ Luzi et al investigated the relation of menin and microRNA 26a (*miR‐26a*), which had a negative post‐transcriptional control on SMAD1. Menin activated *miR‐26a* expression by occupying its promoter. *MEN1* inhibition by siRNA resulted in downregulation of *miR‐26a* and upregulation of SMAD1 protein in osteoblastic‐differentiated human adipose tissue‐derived stem cells (hADSCs), which might explain the suppression role of menin during late osteogenic differentiation.^52^, ^53^ These results indicated that menin positively regulated the early commitment of multipotential mesenchymal stem cells into osteoblast lineage, but inhibited maturation of differentiated osteoblasts.

Kanazawa et al. found that osteoblast‐specific deletion of *Men1* in *Men1* ^*f/f*^ *;Bglap‐Cre* mice showed a reduction of osteoblasts numbers and significant decreases of bone mass and volume in both trabecular and cortical bones. *Men1*‐deficient calvarial osteoblasts exhibit impaired mineral apposition and reduced transcriptional responsiveness to BMP2, leading to downregulation of osteogenesis‐related genes expression.^54^ Unexpectedly, osteoblast‐specific *Men1* knock‐out mice also showed remarkable deficiency in osteoclastogenesis. On the contrary, overexpression of menin specifically in osteoblasts driven by the 2.3‐kb *Col1a1* promoter increased bone mass accumulation but did not affect osteoclast differentiation in vivo.^54^ Similarly, Liu et al. demonstrated that specific deletion of *Men1* in the osteoblast lineage using *Men1* ^*f/f*^ *;Runx2‐Cre* and *Men1* ^*f/f*^ *;Osx‐Cre* mice displayed strong decreases of trabecular bone mass resembling osteoporosis. However, they found no significant changes in osteoblast number and osteoblast function in young‐ and middle‐aged mice lacking menin in the osteoblast lineage. Menin deficiency specifically in osteocytes upregulated the expression of C‐X‐C motif chemokine 10 (*Cxcl10*) and led to enhanced osteoclastogenesis, suggesting menin modulated osteocyte–osteoclast crosstalk in osteoporosis.^55^ Further study showed that aged *Men1* ^*f/f*^ *;Runx2‐Cre* mice displayed lesion of ossifying fibroma (OF) in mandibular bone, with elevated levels of early osteoblast differentiation markers such as ALPL, COL1A1, RUNX2, and OSX. The protein levels of the late osteoblast differentiation marker OCN, however, remained unchanged. The jaw bone‐derived primary mesenchymal stromal cells isolated from the OF tumour (OFMSCs) showed a significant reduction of *Men1* mRNA level and reduced ALP activity and mineralization. These results suggested *Men1*‐dificient OFMSCs were arrested at preosteoblastic differentiation stage, leading to the disordered bone formation in OF lesion.^56^

MLL3 (KMT2C) and MLL4 (KMT2D) are homologues of a Drosophila H3K4 monomethyltransferase, Trithorax‐related (Trr).^57^ Human MLL3/4 COMPASS‐like complex is composed of WRAD, KDM6A (also named as UTX), NCOA6, PAGR1, PAXIP1, and either MLL3 or MLL4, functioning as the major methyltransferase mediating H3K4me1 at enhancer elements.^58^, ^59^ It has been demonstrated that in differentiating MC3T3 cells, WDR5 and KDM6A are both required for transcription of *Runx2/p57*. In addition, MEN1, MLL2, and MLL3 (but not MLL1 or MLL4) bind to *Runx2 P1* promoter, regulating the H3K4me3 state that promotes and sustains the expression of this bone‐master gene in osteoblasts.^34^, ^60^ KDM6A and MLL4 are required for the recruitment and binding of CBP/p300 on enhancers, resulting in an active state decorated with H3K27ac and H3K4me1.^61^, ^62^ Mutations of KDM6A and MLL4 cause Kabuki syndrome, a rare developmental disorder that exhibit systemic defects including craniofacial dysmorphism, growth retardation, and intellectual disability.^63^ A mouse model with a heterozygous mutation in the SET‐domain of MLL4 exhibited similar defects as shown in human Kabuki syndrome, especially a skeletal growth retardation phenotype including shortened long bones and brachycephaly of skulls.^64^, ^65^ Histology analysis of an expansion of growth plate indicated disrupted endochondral ossification. In vitro experiments of murine chondrogenic cell line ATDC5 which had biallelic deletions of *Mll4* showed precocious chondrocytes differentiation. Mechanistically, *Mll4* deletion decreased H3K4me3 modifications on the short stature homeobox 2 (*Shox2*) gene, leading to increased expression of *Sox9* and inhibition of chondrocytes transdifferentiation to osteoblasts.^65^ In contrast, a CRISPR‐Cas9‐mediated truncated mutation of *MLL4* in hTERT‐immortalised human adipose‐derived MSCs showed significantly impaired chondrogenic differentiation and partially impaired osteogenesis, which could be rescued by inhibition of the nuclear mechanosensor, ATR serine/threonine kinase.^66^ Another report demonstrated that in neural crest cells (NCC)‐specific *Mll4* knockout mice, which was generated by *Wnt1‐Cre*, the hypertrophic chondrocyte differentiation and bone deposition in structures of cranial base were significantly decreased, representing a deficiency in endochondral ossification.^67^ The inconsistency of above‐mentioned findings may be ascribed to different knockout strategies and cell types. Further researches are required to dig out the precise functions of MLL4 in craniofacial development.

KDM6A belongs to the H3K27me3 demethylase KDM6 family which also contains lysine demethylase 6B (KDM6B), antagonising the PcG repressive H3K27 methyltransferase EZH2.^68^ Studies have shown that KDM6A is a positive regulator in MSCs lineage commitment and maturation. Retroviral‐mediated *KDM6A* overexpression in human MSCs promoted osteogenesis by activating expression of osteogenic genes and inhibited adipogenesis, whereas *KDM6A* knockdown by siRNA had the reverse effect.^69^ The functions of KDM6A were confirmed by silencing *Kdm6a* in osteoblast cells, resulting from the increased level of H3K27me3 on the promoter regions of Runx2 and Osx.^70^ Furthermore, KDM6A attenuated the enrichment of H3K27me3 on the promoter of Wnt inhibitor Dickkopf‐1 (*Dkk1*) caused by glucocorticoid treatment and was indispensable in averting glucocorticoid‐impaired osteogenesis. Mice treated with glucocorticoid and KDM6A inhibitor GSK‐J4 exhibited a reduction of bone mineral density and trabecular bone loss.^62^ A recent study revealed that *KDM6A* expression was upregulated in osteogenic human periodontal ligament stem cells (hPDLSCs), along with increased *ALPL*, *RUNX2*, and *OPN* expression. As a target of *miR‐153‐3p*, *KDM6A* overexpression reversed the microRNA's inhibitory effect on the osteogenic differentiation of hPDLSCs.^71^

It is known that H3K27me3 levels are regulated during MSCs chondrogenesis. Yapp et al. investigated the role of KDM6A and KDM6B in chondrogenic human bone marrow‐derived MSCs. The expression of *KDM6B* instead of *KDM6A* was increased in chondrogenic differentiation. Knockdown of *KDM6A* and *KDM6B* by siRNA inhibited expression of chondrogenic‐related markers such as aggrecan (*ACAN*), collagen type II alpha 1 chain (*COL2A1*), collagen type X alpha 1 chain (*COL10A1*) and SRY‐box transcription factor 9 (*SOX9*), though *KDM6A* knockdown had a lesser effect. Demethylase inhibitor GSK‐J4 treatment also showed reduced total collagen and glycosaminoglycan (GAG) during MSCs chondrogenesis.^72^ Similarly, KDM6A was required in chondrogenic differentiation of human PDLSCs. Deletion of *KDM6A* via shRNA repressed proteoglycans and collagen formation in both monolayer and micromass culture of hPDLSCs. The mRNA levels of *SOX9*, *COL2A1* and *ACAN* were decreased in *KDM6A*‐deleted hPDLSCs, and H3K27me3 was increased at *SOX9* promoter. The EZH2 inhibitor EPZ‐6438 decreased H3K27me3 level and rescued the impaired chondrogenic potential, suggesting the dynamic balance of H3K27 methylation is an important facet in the regulation of stem cell chondrogenesis.^73^ When cocultured with BMSCs under hypoxia condition, articular cartilage chondrocytes (ACCs) showed enhanced chondrogenic differentiation with upregulation of *Kdm6a* and *Sox9* expression. Treatment with cocultured BMSCs and ACCs via knee joint cavity injection on OA rats lessened the cartilage lesions, which might reveal a promising direction for OA treatment.^74^

### 3.2 SWI/SNF COMPLEXES

In recent years, SWI/SNF complexes have gained much attention for their essential roles in gene expression regulation, chromatin modification maintaining, and DNA repair.^75^ As ATP‐dependent chromatin remodelers, SWI/SNF complexes directly bind DNA with high affinity and yield chromatin access via repositioning or removing nucleosomes, exposing binding sites for proteins and RNAs such as transcription factors and RNA polymerases.^76^ Human SWI/SNF complexes consist of approximately 14 subunits with either BRG1 or BRM as an ATPase.^14^ Other than the strong correlation between mutations of SWI/SNF genes and cancer,^75^, ^77^ more and more studies have demonstrated that these genes also participate in the regulation of development and tissue differentiation. Herein, we mainly focus on the functions of BRG1 and BRM in skeletal development and osteoblast lineage commitment.

The two independent ATPases have distinct roles in mammalian development: *Brm*‐null mice developed normally to adulthood in contrary to the early embryonic lethality of *Brg1*‐null mice.^78^, ^79^ Analysis of BMSCs obtained from *Brm*‐null mice showed significant increases in *Alpl* and *Fgfr2* expression, but the level of RUNX2 and OSX remained unchanged compared to wildtype, suggesting that *Brm* deletion merely mediated the early commitment of mesenchymal stem cells to the osteoblast lineage rather than induced differentiation into mature osteoblast. Moreover, adipogenesis was impaired in *Brm*‐deleted C3H10T1/2 cells as well as *Brm*‐null BMSCs. *Brm*‐null mice showed a phenotype of adiposity reduction in the bone marrow and resistance to age‐related osteoporosis.^80^

Studies by Young et al. assessed the link between SWI/SNF complex subunits and BMP2‐induced osteoblast differentiation. *Brg1* expression was confirmed in the developing skeleton and primary osteoblasts. Transgenic NIH‐3 T3 cells expressing a mutant BRG1 protein showed inhibited BMP2‐induced expression of alkaline phosphatase (APase), indicating that SWI/SNF chromatin remodelling activity is essential for osteogenic lineage induction.^81^ It has been established that BRG1‐containing SWI/SNF complex was recruited by CCAAT/enhancer‐binding protein β (C/EBPβ) to *Bglap* promotor. The ATPase activity of SWI/SNF is required for *Bglap* transcription.^82^ Similarly, BRG1 and BRM were found to be enriched at the *Osx* promoter in osteogenic‐differentiated C3H10T1/2 cells and the catalytic activity of SWI/SNF is required for *Osx* expression.^83^ However, the transcription activation of *Runx2/p57* was independent of SWI/SNF complexes activity as observed in C2C12 cells.^84^

BRM was required for glucocorticoids to get access to the promoters of osteogenesis favoured or adipogenesis inhibiting genes such as *Bglap*, *Fasl*, and *Per3*. BRM deficient MC3T3 cells showed enhanced osteogenic differentiation resisting the repressive effect of dexamethasone. These results indicate that glucocorticoid‐mediated inhibition of osteogenic genes is highly dependent on BRM‐SWI/SNF complexes.^85^ *Brm* and *Brg1* gene depletions in MC3T3‐E1 cells have shown that *Brg1* deficiency impeded osteogenic differentiation, while disruption of BRM accelerated mineralization accompanied higher expression levels of osteogenic markers. BRG1‐specific SWI/SNF complexes were required for activation of tissue‐specific genes. On the contrary, BRM‐containing complexes were found to associate only with the repressed promoter along with the corepressor HDAC1.^86^, ^87^ The SWI/SNF members polybromo 1(PBRM1), AT‐rich interaction domain 2 (ARID2), and bromodomain containing 7 (BRD7) are PBAF complexes specific components. A recent report showed that knockdown of *Pbrm1*, *Arid2*, and *Brd7*, respectively, impaired BMP‐Smad1/5/8‐signalling‐induced expression of osteogenic early responsive genes including *Alpl*, *Osx*, *Runx2*, and attenuated long‐term osteogenesis in OP9 murine MSC cell line and human BMSCs. Mechanistically, co‐localization of PBRM1/ARID2/SMARCC1/EP300 and H3K27ac was found on the transcription start site (TSS) of *Alpl*, *Bmpr1b*, and *Tgfbr2* genes, indicating a direct role of PBRM1/PBAF in transcriptional activation.^88^

The SWI/SNF‐mediated chromatin remodelling mechanism has been proved to exist in cartilage tissue. 11 genes of SWI/SNF complexes including BRG1 and BRM were identified by comprehensive mapping of human cartilage‐expressed transcripts.^89^ During BMP2‐induced chondrogenic differentiation of C3H10T1/2 cells, the expression of fibroblast growth factor receptor 3 (*Fgfr3*) was upregulated by transcription factor SP1. The regulatory mechanism of this process was revealed as BRG1 exposed SP1 binding sites at *Fgfr3* promoter.^90^

### 3.3 ASH1L

Absent, small, or homeotic disc1 like (ASH1L) is another member of the Trithorax group containing a SET domain and is known as an epigenetic transcriptional activator counteracting Polycomb repression.^91^ A recent study revealed the potential role of ASH1L in the epigenetic regulation of MSCs differentiation. Levels of ASH1L were reduced in mice osteoporosis models as well as human osteoporotic samples, indicating a positive correlation of *Ash1l* expression with bone mass. *Ash1l* knockdown suppressed osteogenic and chondrogenic differentiation of C3H10T1/2 cells via decreasing H3K4me3 deposition on the promoter region of *Hoxa10*, *Osx*, *Runx2*, and *Sox9* genes. These results hint the role of ASH1L in osteogenesis and chondrogenesis of MSCs is dependent on its HMTase activity.^92^ Meanwhile, it has been demonstrated that ASH1L occupied transcriptional regions of abundant active genes including the *HOX* genes and was responsible for H3K4 trimethylation.^93^ Another group of studies reported that mammalian ASH1L specifically mono‐ or di‐methylated histone H3K36 based on experiments using nucleosomes as substrates.^94^, ^95^ The controversial observation of its HMTase activity raises the possibility that ASH1L may have an unrevealed role in transcription regulation and stem cell fate determination.

### 3.4 CBP/p300

The Trx complex named TAC1 in Drosophila contains CBP which interacts with Ash1 and confronts Polycomb silencing.^96^ The mammalian CBP and its paralogue p300, encoded by *CREBBP/EP300* genes, exhibit HAT activity that is responsible for H3K27 acetylation (H3K27ac).^22^ As important transcriptional co‐activators, CBP and p300 regulate the expression of a broad range of genes, especially transcription factors, that are involved in cell proliferation, differentiation and other cellular processes.

Homozygous null mutations of CBP or p300 in mice cause early embryonic lethality, and *Crebbp* heterozygous mice show various tissue defects involving the skeletal, haematopoietic and nervous system. It has been found that heterozygous *Crebbp*‐deficient mice showed a significant decrease in trabecular bone volume mainly related to osteoclastogenesis of bone marrow stromal cells since the mineral apposition or bone formation rates are unperturbed.^97^ In humans, monoallelic mutations in either *CREBBP* or *EP300* cause the Rubinstein‐Taybi syndrome, which is autosomal dominant inheritance and exhibits congenital abnormalities such as distinctive facial features, skeletal dysplasia, and intellectual disability.^98^, ^99^

The mechanism of CBP/p300 in gene expression activation has been well established. CBP/p300 associates with gene enhancer and TSS regions, bind to various transcription factors bridging them to RNA polymerase II, and relax the chromatin structure at target gene promoters via their HAT activity.^100^, ^101^ Over the past two decades, it has been demonstrated that CBP, especially p300, has strong correlations with osteogenic‐specific genes including *RUNX2*, *OCN*, *OSX*, and matrix metallopeptidase 13 (*MMP13*).^102^, ^103^, ^104^ Studies have also revealed that CBP/p300 have an integral role during MSCs chondrogenesis. CBP/p300 reportedly enhanced the transcriptional activity of SRY‐box transcription factor 9 (*SOX9*), which is considered a master regulator in chondrocyte differentiation. SOX9, SMAD3, and p300 formed a transcription complex on the SOX9‐binding‐site of *COL2A1* gene enhancer region, and facilitated *COL2A1* expression in human MSCs.^105^, ^106^, ^107^ Additionally, it was established that p300 regulated the expression of chondromodulin (*CNMD*), a cartilage specific protein, in human MSCs. The PcG member YY1 transcription factor repressed chondromodulin expression in undifferentiated MSCs by recruiting histone deacetylase HDAC2. As a co‐activator, p300 is associated with SP3 transcription factor and bind the core‐promoter region of *CNMD* gene. The combination of siRNA inhibition of YY1 and forced expression of p300 and SP3 markedly induced chondromodulin expression.^108^ In summary, CBP/p300 have crucial functions in transcriptional regulation of linage‐specific genes in MSCs.

## 4 CONCLUSIONS

As important components of epigenetic regulation, TrxG proteins mediate transcriptional activation via histone methylation, acetylation, and modifications on chromatin structure. This paper summarises the regulatory roles of TrxG proteins in MSCs osteogenic and chondrogenic differentiation, as shown in Table 1. Targeting of TrxG proteins may hold great promise in stem‐cell‐based epigenetic therapies regarding bone and cartilage regeneration. Notably, dysregulation of TrxG proteins also marks a strong correlation with malignancy,^109^ calling for further exploration on the precise regulation of their regenerative function. Moreover, the majority of the current studies mostly focused on individual subunits rather than the complexes, while several TrxG proteins including COMPASS and SWI/SNF function in the form of multiprotein complexes. The simultaneous intervention of multiple genes in complexes is not a solution. Because their enzymatic activities and interactions differ in different complexes or cell types, the inhibition or knockdown of a single subunit may change the functions of other components in the complex. Further studies are needed to probe into the function of complexes as a whole and the interactions between various components. To achieve this, it is important to figure out the key connecting link in the complexes. Ideally, mutation of key sites or small molecule chemical agent targeting towards the link site would tear the complexes apart and thus cancel the complexes' functions. Taken together, the review focused on the studies of TrxG proteins in regulation of osteogenesis and chondrogenesis. Nevertheless, several scientific issues above mentioned need to be addressed, TrxG proteins act as one of the most promising epigenetic regulators in bone and cartilage regenerations.

**TABLE 1:** Role of TrxG on osteogenic and chondrogenic differentiation

TrxG complex subunits	Targeted genes	Cells	Functions in vitro	Functions in vivo	Ref.
WRAD core subcomplex (WDR5/RBBP5/ASH2L/DPY30)					
WDR5	*WNT1MYC RUNX2*	MC3T3‐E1 cells	Promote osteogenic differentiation	—	33
WDR5	*RUNX2*	C2C12 cells	Promote osteogenic differentiation	—	34
WDR5	*IBSP*	Mice calvarial osteoblasts	Promote osteogenic differentiation	—	35
WDR5	*CTNNB1*	Human bone marrow mesenchymal stem cells (hBMSCs)	Interact with LncRNA HOTTIP and promote osteogenic differentiation	—	36
WDR5	*TWIST1*	MC3T3‐E1 cells	Promote osteogenic and chondrogenic differentiation	Overexpressions of WDR5 in mice perichondrium cause larger skeleton and increased hypertrophic chondrocyte layer size	37, 38, 39
MLL1/2 COMPASS‐like (MLL1/MLL2/Menin/HCFC1)					
MLL1	*HOXD4 HOXC8*	—	—	Mutant MLL1 causes skeletal defects such as vertebral column and sternal malformations	44
MLL1/2	*DLX3*	Human dental follicle stem cells (DFCs)	Promote osteogenic differentiation		45
Menin	—	—	—	*Men1* knockout causes embryonic lethality and craniofacial developmental defects	47
Menin	*SMAD1/5 RUNX2 TGFB1 SMAD3*	C3H10T1/2 cells; ST2 cells; MC3T3‐E1 cells	Promote early osteogenic differentiation; inhibit late osteoblast maturation	—	48, 49
Menin	*LEF1 CTNNB1*	C2C12 cells	Promote osteogenic differentiation	—	50
Menin	*JUND*	MC3T3‐E1 cells	Suppress osteoblasts maturation	—	51
Menin	*miR‐26a*	Human adipose tissue‐derived stem cells (hADSCs)	Inhibit SMAD1 expression in late osteogenic differentiation	—	52, 53
Menin	—	—	—	Osteoblast‐specific *Men1* knockout causes decreased bone mass and volume in trabecular and cortical bone	54
Menin	—	—	—	*Men1* deletion causes osteoporosis‐like phenotype	55
Menin	—	—	—	*Men1* deletion causes ossifying fibroma lesion in mandibular bone	56
MLL3/4 COMPASS‐like (MLL3/MLL4/KDM6A/NCOA6/PAGR1/PAXIP1)					
MLL3	*RUNX2/P1*	MC3T3 cells	Promote osteogenic differentiation	—	60
MLL4 KDM6A	—	—	—	Mutations of MLL4 and KDM6A cause Kabuki syndrome with skeletal growth retardation and craniofacial dysmorphism	62, 63
MLL4	*SHOX2*	ATDC5 cells	Deletion of MLL4 promotes precocious chondrocytes differentiation, inhibit chondrocytes transdifferentiation to osteoblasts	Mutations of MLL4 cause shortened long bones, brachycephaly of skulls and expansion of growth plate	64, 65
MLL4	*ATR*	hTERT‐immortalised human adipose‐derived MSCs	Deletion of MLL4 inhibits chondrogenic and osteogenic differentiation	—	66
MLL4	—	—	—	NCC‐specific *Mll4* knockout mice show facial dysmorphism	67
KDM6A	*RUNX2 OSX*	hMSCs; MC3T3‐E1 cells	Promote osteogenic differentiation	—	69, 70
KDM6A	*DKK1*	Immortalised murine osteogenic progenitor cell	Attenuate glucocorticoid‐impaired osteogenesis	—	62
KDM6A	*miR‐153‐3p*	Human periodontal ligament stem cells (hPDLSCs)	Promote osteogenic differentiation	—	71
KDM6A	*SOX9*	hPDLSCs	Promote chondrogenic differentiation	—	73
KDM6A		BMSCs; Articular cartilage chondrocytes (ACCs)	Promotechondrogenic differentiation	—	74
SWI/SNF complexes					
BRM	—	BMSCs C3H10T1/2 cells	Suppress early osteogenic differentiation	*Brm* knockout causes reduced bone marrow adiposity and resistance to age‐related osteoporosis	80
BRG1	*BGLAP*	ROS17/2.8 osteoblastic cells	Promote osteogenic differentiation	—	82
BRG1BRM	*OSX*	C3H10T1/2 cells	Promote osteogenic differentiation	—	83
PBRM1ARID2SMARCC1	*ALPL BMPR1B TGFBR2*	OP9 murine MSC cell line; hBMSCs	Promote osteogenic differentiation	—	88
BRG1	*FGFR3*	C3H10T1/2 cells	Promote chondrogenic differentiation	—	90
ASH1 (ASH1L/CREBBP/EP300)					
ASH1L	*HOXA10 OSX RUNX2 SOX9*	C3H10T1/2 cells; hBMSCs	Promote osteogenic and chondrogenic differentiation	—	92
CREBBP	—	—	—	*Crebbp* ^+/−^ mice show trabecular bone volume decrease	97
CREBBPEP300	*RUNX2 BGLAP OSX MMP13*	—	Promote osteogenic differentiation	—	102, 103, 104
EP300	*COL2A1*	hMSCs	Promote chondrogenic differentiation	—	105, 107
EP300	*CNMD*	hMSCs	Promote chondrogenic differentiation	—	108

## CONFLICT OF INTEREST

The authors declare no conflict of interest.

## AUTHOR CONTRIBUTIONS

Q.M. C.S. and B.Y. collected the related paper and drafted the manuscript. C.S. and Y.S. revised the manuscript. L.Y. designed the review and revised the manuscript. All authors read and approved the final manuscript.

## Data Availability

The data sharing is not applicable to this article.
